# Stress Assessment in Parents of Children With Autism Spectrum Disorder: A Prospective Case-Control Study

**DOI:** 10.7759/cureus.70438

**Published:** 2024-09-29

**Authors:** Kritika Negi, Vasu Saini, Shruti Kumar, Utkarsh Sharma, Nidhi Elizabeth Jacob

**Affiliations:** 1 Pediatrics, Shri Guru Ram Rai Institute of Medical and Health Sciences, Dehradun, IND; 2 Internal Medicine, Government Medical College Mahasamund, Kharora, IND

**Keywords:** autism spectrum disorder (asd), developmental disorders, emotional impact, neurodevelopmental assessment, parenting stress

## Abstract

Background: Autism spectrum disorder (ASD) is a disorder that is manageable but has no cure. Therefore, it is associated with parental stress, which is often not given importance. Early identification and holistic management of children with ASD are needed.

Methods: This prospective case-control study, conducted over 18 months, included 111 newly diagnosed autism cases (ages 2-9) from the Child Development Clinic at Shri Mahant Indiresh Hospital, Dehradun, India, diagnosed as per the International Clinical Epidemiology Network (INCLEN) criteria (DSM-V). Controls were 99 typically developing children from a well-baby clinic. Data collection involved administering the Autism Parenting Stress Scale and a predesigned questionnaire to both cases and controls. Parents with psychiatric or mental health issues were excluded.

Results: Parents of children with autism exhibited significantly higher stress scores compared to parents of typically developing children. In our study, out of a total of 210 subjects, 52.9% were diagnosed with autism, while 47.1% were controls. Cases were analyzed based on household and socioeconomic background. It was found that 94.6% of the cases showed significant stress levels.

Conclusion: ASD significantly impacts parental stress, particularly among families from higher socioeconomic backgrounds and metropolitan areas. This study emphasizes the need for targeted support and interventions to address elevated stress levels and improve family dynamics, especially for parents of higher-functioning children.

## Introduction

Autism spectrum disorder (ASD) is a complex neurodevelopmental illness characterized by repetitive/restricted behavior patterns, activities, and interests, as well as consistently poor verbal and nonverbal communication and social interaction. It usually first appears in early childhood [1]. Along with a number of comorbidities such as motor disability, attention disorders, externalizing behaviors like violence, feeding difficulties, obesity, mood disorders, epilepsy, and sleep disorders, these children struggle greatly with social contact and emotional reciprocity.

Parental stress is an unfavorable psychological response to the demands of parenthood, where parents find it difficult to provide for their children [2]. These parents deal with a variety of stressors that are linked to the child's traits and deficiencies, the severity of the illness, the parents' individual traits, the support system in the community, and the family's coping mechanisms. It can be very difficult for parents to accept that their child has a chronic illness that will affect them for the rest of their lives. Parenting stress experienced by parents of children with ASD in the Southeast Asia (SEA) region has been linked to multiple factors [3]. These include the child's degree of symptoms, social support, financial problems, and marital and family problems.

In the end, stress makes parenting more challenging since it can lead to a decrease in parental involvement, focus, tolerance, and patience with the child. It would be easier for a parent to care for a child with autism or other neurodevelopmental issues if they are mentally tough and strong.

## Materials and methods

Study design, study period, and study participants

This was a prospective case-control study conducted over 18 months. Patients were enrolled in the Pediatrics OPD and Child Development Clinic (CDC) Unit after their child was diagnosed with autism. A total of 111 newly diagnosed autism cases (ages 2-9) from Shri Mahant Indiresh Hospital, Dehradun, India, were assessed using the International Clinical Epidemiology Network (INCLEN) criteria (based on DSM-V) autism tool and Indian Scale for Assessment of Autism (ISAA). Kuppuswamy's classification was used to define socioeconomic status (SES). The study also included 99 typically developing children, matched for age and sex, as controls. Comprehensive assessments were performed, and early intervention was recommended for the autism cases. Data collection involved face-to-face interviews and was analyzed using MS Excel (Microsoft Corporation, Redmond, Washington, United States) and Stata 13 (StataCorp LLC, College Station, Texas, United States).

Study procedure and data collection

After the diagnosis was revealed to the parents, they were subjected to the Autism Parenting Stress Scale and a questionnaire-based predesigned proforma. The same scale was also applied to 99 controls of the same age group who were typically developing children visiting the well-baby clinic.

Inclusion Criteria

The study included parents of children who were recently diagnosed with ASD. Our inclusion criteria for the diagnosis of ASD are based on DSM-V, which focuses on deficits in social-emotional reciprocity and difficulties in developing relationships. The diagnosis also includes repetitive or stereotyped speech and motor behaviors. Additionally, these children exhibit strict adherence to routines, ritualistic behaviors, and intense, fixated interests that are abnormal in focus and intensity.

Exclusion Criteria

Parents with psychiatric or mental health problems (if they are on medication or undergoing psychotherapy) and children with comorbidities such as developmental delay (including motor impairment), attention problems, externalizing behaviors (such as aggression), feeding issues, obesity, affective disorders, epilepsy, and sleep dysfunction were excluded from the study.

Ethical consideration and statistical analysis

The data collected were analyzed utilizing statistical software, specifically MS Excel and Stata 13. Approval for the study was secured from the Shri Guru Ram Rai Institute of Medical and Health Sciences Institutional Ethics Committee (approval number: SGRR/IEC/66/21). Information from parents was obtained through structured interviews, employing a questionnaire developed using MS Word and MS Excel. Statistical analyses were conducted using IBM SPSS Statistics for Windows, Version 21.0 (Released 2012; IBM Corp., Armonk, New York, United States). A p-value of less than 0.05 was considered statistically significant. Statistical tests such as t-tests will be applied wherever needed to compare results.

## Results

**Figure 1:**
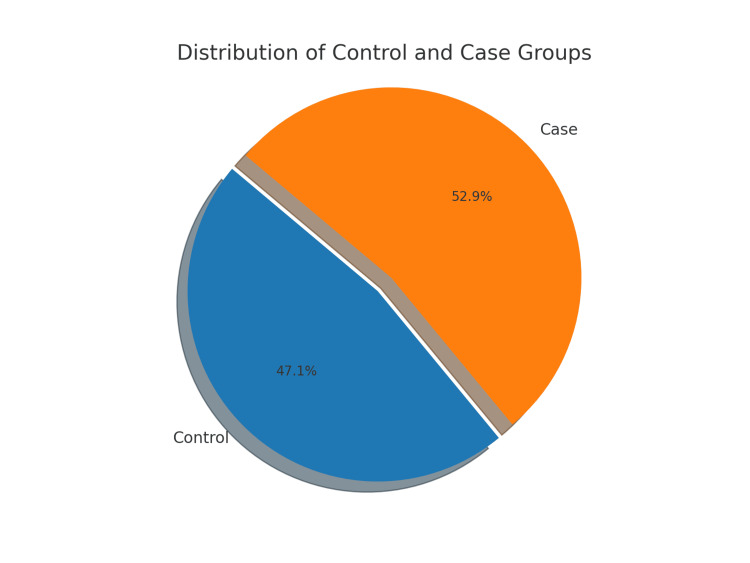
Proportion of participants by group (control vs. case)

Frequency and percentage were obtained to summarize qualitative or categorical data. The association between autism and background variables, such as area, SES, and Parenting Stress Index (PSI) score, was tested using Pearson's chi-squared test. The relationship between the PSI score and ISAA was analyzed using the K-P (Kendall-Pearson) correlation coefficient. The significance level for the study was set at 5%, indicating that a p-value of less than 0.05 was considered statistically significant. In our study, 111 cases with autism (52.9%) and 99 controls (47.1%) were enrolled (Table 1, Figure 1).

**Table 1:** Distribution of participants by group (control vs. case)

Group	Frequency	Percentage
Control	99	47.1
Case	111	52.9
Total	210	100

**Figure 2:**
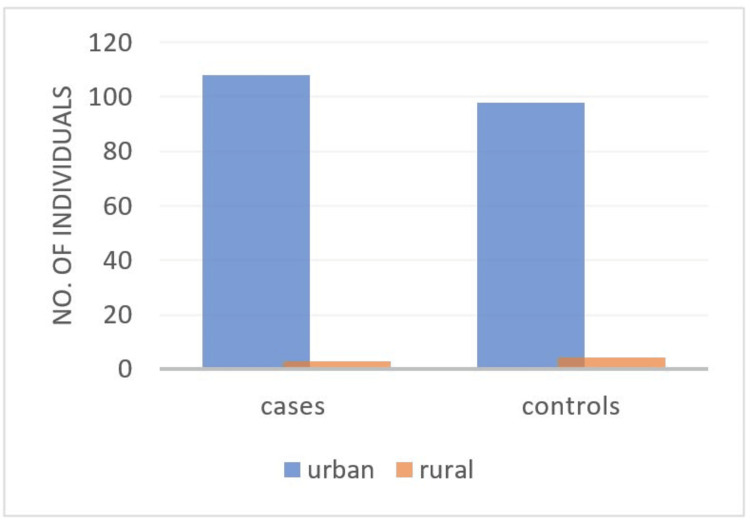
Comparison of participant distribution by area (urban vs. rural) in control and case groups

Of the total number of autism cases, 97.3% belonged to urban households, while 99% of the controls belonged to urban households (Table 2, Figure 2). The chi-squared test yielded a value of 0.802 with a p-value of 0.370. This result indicates that there is no statistically significant association between the area (urban vs. rural) and group status (control vs. case) in this study, as the p-value is greater than the conventional threshold of 0.05.

**Table 2:** Distribution of participants by area (urban vs. rural) in control and case groups Statistical analysis was performed using Pearson's chi-squared test. The test yielded a chi-squared value of 0.802, with a p-value of 0.370, indicating no statistically significant association between the variables.

Area	Control	Autism cases	Total
Urban	98	108	206
	99%	97.3%	98.1%
Rural	1	3	4
	1%	2.7%	1.9%
Total	99	111	210
	100%	100%	100%

**Figure 3:**
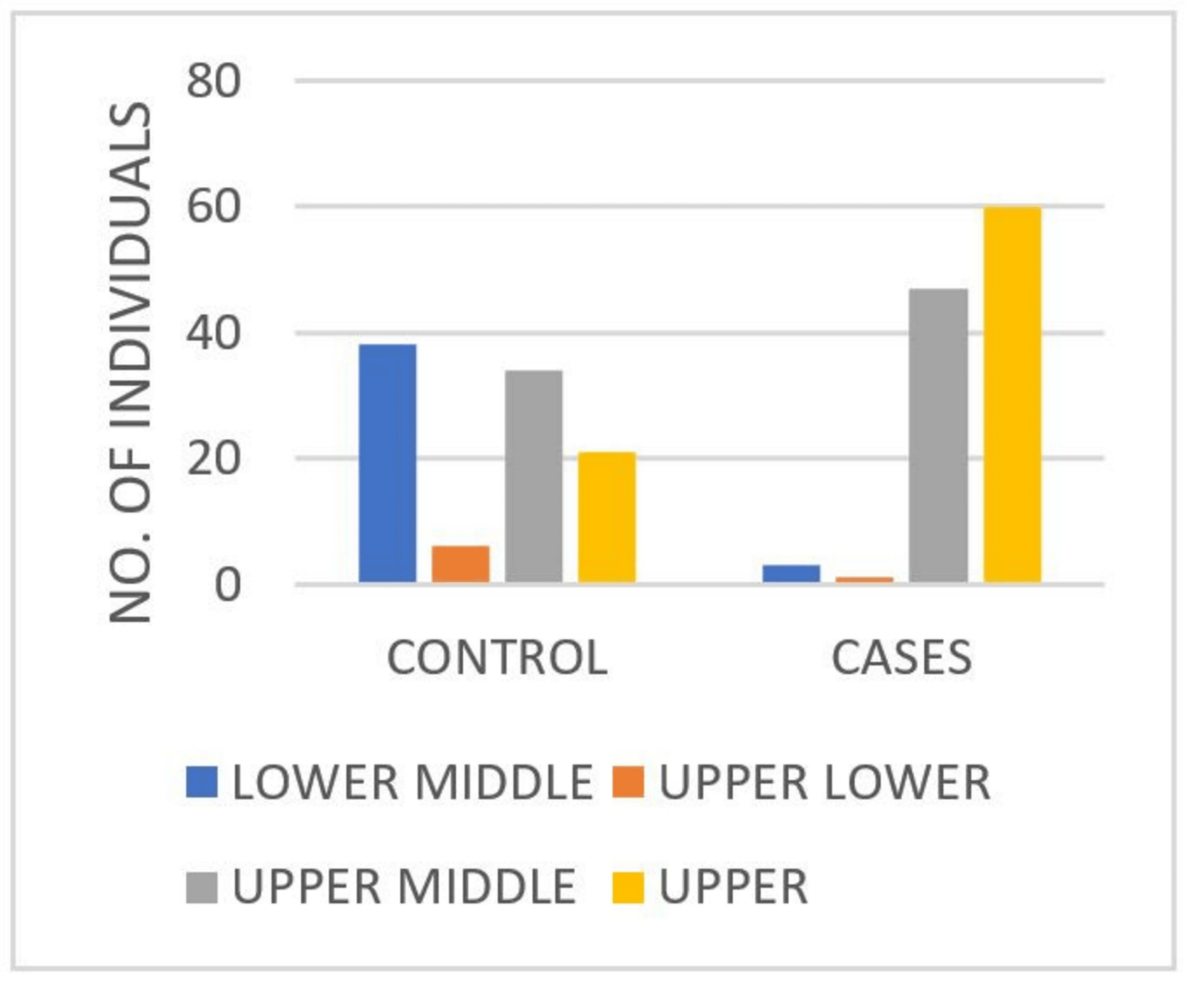
Comparison of SES between control and case groups SES: socioeconomic status

Among the cases, 54.1% belonged to the upper class while only 2.7% were from the lower middle class. In contrast, only 21.2% of the controls were from the upper class and 38.4% were from the lower middle class with a significant p-value (Table 3, Figure 3). This result indicates a highly significant association between the variables tested. The chi-squared statistic of 53.804 suggests a strong deviation from the expected frequencies, and the p-value of less than 0.001 confirms that this deviation is statistically significant. This implies that the observed data are unlikely to have occurred by chance alone.

**Table 3:** Distribution of participants by SES in control and case groups Statistical analysis was conducted using Pearson's chi-squared test. The test yielded a chi-squared value of 53.804, with a p-value of <0.001, indicating a highly significant association between the variables. SES: socioeconomic status

SES	Control	Case	Total
Lower middle	38	3	41
	38.4%	2.7%	19.5%
Upper lower	6	1	7
	6.1%	0.9%	3.3%
Upper middle	34	47	81
	34.3%	42.3%	38.6%
Upper	21	60	81
	21.2%	54.1%	38.6%
Total	99	111	210
	100%	100%	100%

**Figure 4:**
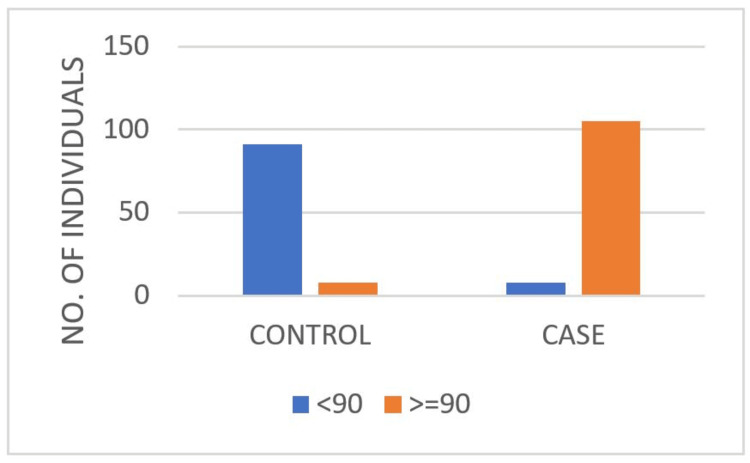
Distribution of PSI scores by group (control vs. case) PSI: Parenting Stress Index

Additionally, 91.9% of the controls showed no significant stress on the PSI scale, compared to 94.6% of the cases, who showed significant stress levels with a significant p-value (Table 4, Figure 4). This result shows a very strong association between the variables being analyzed. The chi-squared statistic of 157.57 indicates a substantial deviation from expected frequencies, and the p-value of less than 0.001 suggests that this deviation is highly statistically significant. This means the observed differences are very unlikely to be due to random chance.

**Table 4:** Distribution of PSI scores across control and case groups PSI measures the stress in the parent-child relationship and considers factors such as the child's distractibility, adaptability, and mood. Statistical analysis was conducted using Pearson's chi-squared test. The test yielded a chi-squared value of 157.57, with a p-value of <0.001, indicating a statistically significant result. PSI: Parenting Stress Index

PSI score	Group		Total
	Control	Case	
<90	91	6	97
	91.9%	5.4%	46.2%
≥90	8	105	113
	8.1%	94.6%	53.8%
Total	99	111	210
	100%	100%	100%

**Figure 5:**
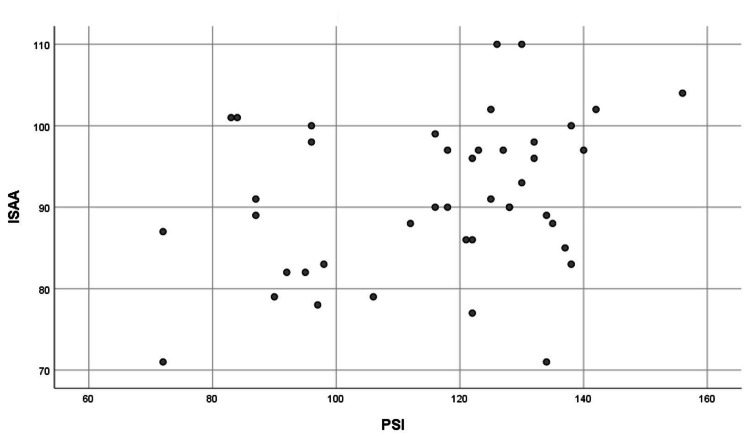
Weak positive correlation between the severity of autism in a child (ISAA score) and parental stress (PSI score) among various SES groups PSI is a tool used to assess the level of stress experienced by parents in raising their children. Higher PSI scores indicate greater levels of parenting stress. ISAA is a standardized scale used for the assessment and diagnosis of ASD in individuals. It provides a comprehensive evaluation of autism-related symptoms and behaviors. PSI: Parenting Stress Index; ISAA: Indian Scale for Assessment of Autism; ASD: autism spectrum disorder; SES: socioeconomic status

In our study, it was found that there was a weak positive correlation among various socioeconomic status groups (Table 5, Figure 5).

**Table 5:** Correlation between SES and K-P coefficients and significance levels SES and K-P correlation coefficients are statistical measures used to assess the strength and direction of the relationship between two variables. K-P correlation coefficients were calculated to assess the relationship between SES categories and the measured variable. P-values indicate the statistical significance of the correlations, with * denoting significance at the 5% level. SES: socioeconomic status; K-P: Kendall-Pearson

SES	K-P correlation coefficients	P-value
Lower middle (n=2)	-1.000	NA
Upper lower (n=1)	NA	NA
Upper middle (n=34)	0.229	0.193
Upper (n=43)	0.310	0.043*

## Discussion

We evaluated parental stress in parents of children with ASD in the current study. Patients were recruited from the Pediatrics OPD and CDC Unit following a diagnosis of autism in their child. The frequency and percentage distribution of socio-demographic variables were as follows.

In our study, urban areas accounted for 97.3% of the cases and rural areas for 2.7%, while 99% of the cases and 1% of the controls were from urban areas. This indicates that the prevalence of autism is higher in urban than in rural settings. One possible explanation for this could be the low awareness of illnesses like autism in rural areas. Furthermore, we included individuals in our study after the diagnosis was initially disclosed at the beginning of therapy. After receiving a diagnosis, a large number of patients from rural areas returned home, neglected to attend therapy sessions at our center, and were excluded from this study.

The parents of autistic children were divided into four social classes: upper class (54%), upper middle class (42.3%), higher lower class (0.9%), and lower middle class (2.7%). Just 21% of controls were from the upper class, 34% were from the upper middle class, 6% were from the upper lower class, and 38% were from the lower middle class. When comparing the cases and controls based on the family's socioeconomic position, there was a notable difference. This raises the potential that children from higher socioeconomic backgrounds may be more likely to be diagnosed with autism. This has been explained by a number of theories, some of which include better healthcare services, greater awareness of such families, etc. Similar findings were observed in the study "From symptom recognition to diagnosis: children with autism in urban India" by Daley, which discovered that middle-class to upper-class families were the majority of families with autistic children. This finding was probably due to the fact that lower-class families were less likely to have an autistic child diagnosed [4]. A study by Malhi et al., which was carried out in a tertiary care hospital in north India, to "understand the perceived barriers for obtaining a diagnosis and the perspectives and experiences of parents of children with autism," also supported this [5]. The majority of the families with children diagnosed with autism, according to the study's findings, belonged to the upper-middle and upper socioeconomic class (71.4%) and were located in metropolitan areas (89.3%). He et al. conducted a population-based study in China to examine the association between childhood autism and socioeconomic position. Their findings indicated that children from households experiencing socioeconomic disadvantage, as seen by lower family income and education levels, were more likely to have autism [6].

In our study population, the mean age of individuals in the control group was 4.77±2.01 years, while the mean age of cases was 3.74±1.44 years. The average age at which autism cases were first concerned was 2.5 years, in line with previous research. At this age, parents typically become aware of abnormalities in their child's development; delays in the speech and language domains are among the first signs. Similar findings were observed by Daley in her Indian study, where parents claimed that their child's first noticeable difference was approximately 2.1 years old, which is also quite similar to our mean of 2.5 years. Nonetheless, research conducted in Western nations has indicated that the average age at which a concern arises is between 14.9 and 19.1 months [7,8]. This brings us to a worrying observation: it has been noted that Asian families delay seeking medical attention, maybe as a result of ignorance regarding abnormal and developmental habits. Additionally, all children in Western nations undergo developmental screening, which includes the Modified Checklist for Autism in Toddlers (M-CHAT) score, at the age of 18 months. This screening is known to identify many cases of autism. Even though developmental screening is currently recommended by our recommendations, it is still inadequate in our nation. Therefore, it is imperative that pediatricians have a heightened awareness of developmental screening and surveillance during routine visits [9-11]. Our study's typical age at which a patient sought medical attention for the first time was 2.8 years or around three months after exhibiting symptoms.

According to a study by Gong et al., parents of autistic children experienced higher levels of stress overall and in both the child and parent domains [12], compared to parents of typically developing children. In our study, parents of autistic children had a mean PSI score of 116.33, which was higher than that of the typically developing group (61.14). The two PSI domains that displayed higher stress scores in both cases and controls compared to the parents' perception of their own behavior (PSI-a: parent distress domain) were the parents' interaction and expectations of their child (PSI-b: parent-child dysfunctional interaction) and the parents' perception of the child's behavior (PSI-c: difficult child). On the PSI scale, 94.6% of the cases indicated significant stress, compared to 91.9% of the controls who did not. Between the two groups, there was a statistically significant difference. The results of prior studies [13,14], which showed that a significant proportion of parents of children with ASD experience stress at or above the clinical threshold, were supported by the results of this study. The proportion of parents in our study who reported significant stress is higher than in previous studies involving similar families and preschool-aged children, even though we used stricter clinical criteria (e.g., Davis and Carter [15]). The results are consistent with those of Davis and Carter [15], who discovered that parents whose children had just received an ASD diagnosis and were beginning to receive support were the ones under the most stress [15]. In a similar vein, parents of autistic children demonstrated stress scores above the 85th percentile, indicating significant stress levels [16], in a study by Padden and James titled "Stress Among Parents of Children With and Without Autism Spectrum Disorder: A Comparison Involving Physiological Indicators and Parent Self-Reports." Our research emphasizes the importance of having official family support throughout the interim period after a diagnosis before seeking additional help.

## Conclusions

ASD is a neurodevelopmental disorder that places significant stress on parents and families. Given that it is a lifelong diagnosis, parents' emotional and physical suffering may never stop. The degree of stress that a parent experiences affects not only their child but also the parent and the quality of their relationships. Understanding the components of this disorder and its relationship to parental stress is crucial. This study highlights several stress-related factors that must be considered when developing support for families with young children with ASD. The study found that most autistic children were from higher socioeconomic backgrounds (54%) and metropolitan areas (97.3%). Parents typically noticed their child's symptoms at 2.5 years and sought medical attention by 2.8 years. Stress levels were significantly higher among parents of children with ASD (94.6%) compared to parents of generally functioning children (8.1%). Regression analysis of the child's autism severity (based on ISAA scores) and parental stress (based on PSI scores) revealed a weakly positive link among the upper-class socioeconomic group. It would be advantageous to offer parents of children with ASD direct and methodical assistance in addition to the services the child receives. Increasing family participation and inclusion should be the aim of all interventions. If we want to encourage parents to collaborate more, we must customize tactics to meet each parent's specific needs because different aspects of raising children can be stressful for them.
